# Late Mortality and Paclitaxel-Coated Devices: Has the Controversy Finally Come to an End?

**DOI:** 10.1016/j.jscai.2023.100981

**Published:** 2023-07-12

**Authors:** Aishwarya Raja, Eric A. Secemsky

**Affiliations:** a Department of Medicine, Columbia University Medical Center, New York, New York; b Richard A. and Susan F. Smith Center for Outcomes Research in Cardiology, Department of Medicine, Beth Israel Deaconess Medical Center, Boston, Massachusetts; c Division of Cardiology, Department of Medicine, Beth Israel Deaconess Medical Center, Boston, Massachusetts

**Keywords:** drug-coated balloon, paclitaxel, peripheral artery disease

Advances in technology have led to the emergence of endovascular therapy as a primary treatment option for patients with peripheral artery disease (PAD), with the goal of improving quality of life and limb-related outcomes.^1^^,^^2^ Numerous randomized controlled trials (RCTs) and meta-analyses have demonstrated higher patency and lower target lesion failure rates associated with drug-coated devices,^3^, ^4^, ^5^, ^6^ establishing these devices, particularly drug-coated balloons (DCBs) containing the antiproliferative agent paclitaxel, as the first-line treatment for patients with symptomatic femoropopliteal PAD.^7^, ^8^, ^9^

Despite the promising evidence supporting the use of drug-coated devices in the periphery, the summary-level meta-analysis of Katsanos et al^10^ in 2018 generated major ripples in the vascular community. The study found that paclitaxel-coated devices were associated with a higher rate of mortality relative to non–paclitaxel-coated devices at 2 years and through 5 years after treatment. The authors also reported a positive association between increasing paclitaxel doses and absolute risk of mortality. These findings had widespread impact across the regulatory and scientific sectors. The Food and Drug Administration (FDA) convened a Medical Device Advisory Panel in June 2019 to investigate the possibility of a late mortality signal.^11^ After analyzing internal trial data of paclitaxel-coated devices and arriving at similar conclusions, they issued warnings about the possibility of increased mortality associated with these devices despite not establishing a causal relationship and recommended restricting use to high-risk patients only.^12^ It also led to the halting of major clinical trials, such as the Swedish Drug-elution Trial in Peripheral Arterial Disease (SWEDEPAD) and Balloon versus Stenting in severe Ischemia of the Leg-3 (BASIL-3), as well as an overall decrease in the use of drug-coated devices.^13^

**Figure 1:**
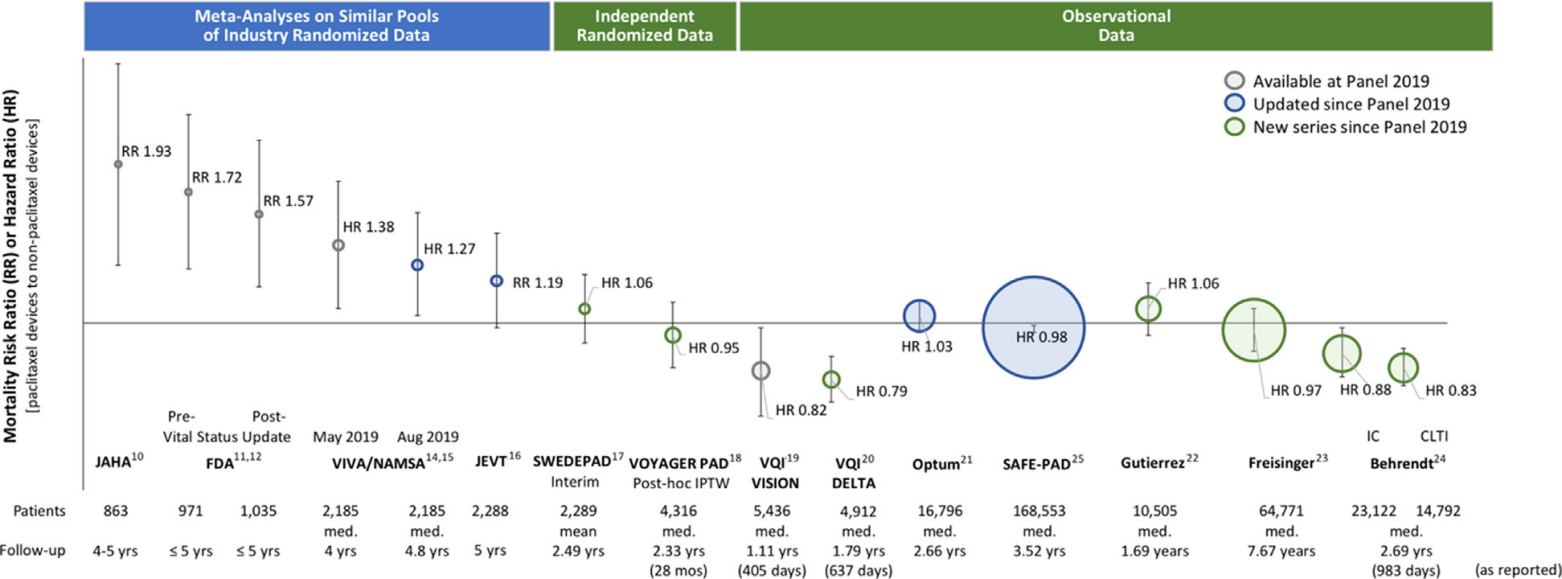
Summary of updated data since the publication of the original meta-analysis. Independent randomized and real-world evidence demonstrates no mortality signal with paclitaxel.

From a methodological standpoint, the study of Katsanos et al^10^ was found to have many limitations. This included the presence of heterogeneous patient populations across the pooled RCTs; a substantial loss to follow-up, withdrawal, and patient cross-over; as well as the lack of an established mechanism to explain the late mortality signal associated with paclitaxel. To address these limitations and further explore the association between paclitaxel and mortality risk in other populations, several studies have since been published, including updated meta-analyses, subanalyses of RCTs, and large observational cohort and registry studies (Figure 1). For example, the Vascular InterVentional Advances (VIVA) physicians group performed a meta-analysis using patient-level data from 8 RCTs of paclitaxel-coated devices approved in the United States. After including expanded follow-up information and reducing missing data, the study showed a significant but attenuated risk of mortality associated with paclitaxel-coated devices up to 4 years.^14^^,^^15^ Additionally, the meta-analysis of Dinh et al,^16^ which included additional trials and a larger sample size, showed no increased risk of mortality associated with paclitaxel-coated devices up to 60 months of follow-up. Unplanned interim and subgroup analyses of the SWEDEPAD^17^ and Vascular Outcomes Study of ASA Along With Rivaroxaban in Endovascular or Surgical Limb Revascularization (VOYAGER PAD)^18^ RCTs, respectively, also failed to demonstrate mortality signals.

To complement these prospective studies, multiple real-world observational studies using the Vascular Quality Initiative registry,^19^^,^^20^ Optum claims,^21^ Veterans Health Insurance claims,^22^ German BARMER health insurance claims,^23^^,^^24^ and Medicare claims^25^ have also failed to detect survival differences among patients treated with paclitaxel-coated and uncoated devices. For instance, the Safety Assessment of Femoropopliteal Endovascular Treatment with Paclitaxel-coated Devices (SAFE-PAD) study (ClinicalTrials.gov identifier NCT04496544), an ongoing prespecified longitudinal safety assessment of paclitaxel-coated devices among Medicare patients with a median follow-up exceeding 5 years, has thus far noted no evidence of long-term harm associated with these devices.^26^^,^^27^

With this context in mind, in this issue of *JSCAI,* the study of Lyden et al^28^ extends the assessment of mortality risk associated with drug-coated devices using pooled individual-level data from the Stellarex DCB trials program. The aim of the study was to evaluate the 5-year all-cause mortality rate among patients treated with the Stellarex DCB compared with uncoated percutaneous transluminal angioplasty (PTA), which is an extension of a prior meta-analysis of the ProspectIve, Randomized, SingLe-Blind, U.S. MuLti-Center Study to EvalUate TreatMent of Obstructive SupErficial Femoral Artery or Popliteal LesioNs With A Novel PacliTaxel-CoatEd Percutaneous Angioplasty Balloon (ILLUMENATE) RCTs that showed no difference in all-cause mortality between treatments through 4 years of follow-up.^29^ An independent third party performed the patient-level meta-analysis that involved pooling all prospective RCTs. Included patients had Rutherford 2-4 symptomatic femoropopliteal disease, and the primary outcome was all-cause mortality.

Overall, the meta-analysis showed no difference in survival between the Stellarex DCB and the PTA arms at 5 years. Importantly, there was no difference in mortality when stratified by terciles of cumulative paclitaxel dose received, further debunking a dose-mortality relationship presented in the original Katsanos meta-analysis. Finally, when examining predictors of death, neither paclitaxel dose nor overall exposure was shown to be significantly associated with future mortality.

Particular strengths of the study include its large sample size and homogenous patient cohort. Importantly, the vital status compliance was 93.8% across the RCTs, which allowed for a more complete assessment of the endpoint (for context, pivotal trials had between 10% and 30% missing vital status data at the time of the initial meta-analysis). Limitations of the study include the inability to quantify the number of patients exposed to paclitaxel in the PTA group due to inconsistently reported repeat revascularization data; potential inaccuracies in calculating the paclitaxel dose due to unmeasured variables like lesion characteristics and blood flow; and the lack of generalizability of the study’s findings to other paclitaxel-coated devices.

So where do we stand now? Since the original Katsanos meta-analysis was published in December 2019, we have more than a dozen independent analyses that have failed to associate paclitaxel exposure and long-term survival. As loss to follow-up has been incrementally addressed, signals of harm have attenuated to the point of nonsignificance, suggesting there was something different about patients who had survival data available versus not when the initial meta-analyses were performed. Drug-coated devices in clinical use have in parallel rebounded in the United States, moving closer to precontroversy rates. Nonetheless, US regulators have maintained the current position that there remains a possibility of harm related to these devices. This has impacted use for some clinicians and institutions across the country, whereas the largest influence of this stance may in fact be with regulatory bodies outside the United States, who often look to the United States for guidance. With the ILLUMENATE trials program of 589 total patients with 5 years of data (the original meta-analysis only had 863 patients at 4-5 years) clearly demonstrating no survival difference, it is difficult to label this specific device as harmful.

The upcoming year will be critical. We will see updated data from pooled patient-level data across all device platforms as well as from other studies including the ongoing Safety Assessment of Femoropopliteal Endovascular treatment with Paclitaxel-coated Devices study. Will this be enough to re-enter into conversations with the FDA about revising the current letter to health care providers and label changes? As of now, it remains unclear, yet the body of work that has been produced as a result of this controversy towers over the original meta-analysis, with consistent signals of safety.
